# Sexual selection for bright females prevails under light pollution

**DOI:** 10.1093/cz/zoaa071

**Published:** 2020-12-28

**Authors:** Christina Elgert, Topi K Lehtonen, Arja Kaitala, Ulrika Candolin

**Affiliations:** 1 Organismal and Evolutionary Biology, University of Helsinki, Helsinki, PO Box 65, 00014, Finland; 2 Tvärminne Zoological Station, University of Helsinki, J.A. Palménin tie 260, Hanko, 10900, Finland; 3 Department of Ecology and Genetics, University of Oulu, Oulu, PO Box 3000, 00014, Finland, 90014

**Keywords:** environmental change, mate choice, mate attraction, reproduction, sexual selection

The functions of sexually selected traits are particularly sensitive to changes in the environment because the traits have evolved to increase mating success under local environmental conditions (Rosenthal and Stuart-Fox 2012). When environmental conditions change, previously reliable signals may become less reliable or harder to detect and evaluate. Because the correct expression, transmission, and interpretation of sexual signals typically influence mate choice outcomes, impediments to sexual signals can change both the strength and the direction of sexual selection (Rosenthal and Stuart-Fox 2012). Artificial light is a major anthropogenic disturbance that is intensifying around the world and has high potential to negatively impact wildlife, for example by hampering the expression and detection of sexual signals. For instance, the bioluminescent signals of fireflies are often inhibited or obscured by artificial illumination (Rosenthal and Stuart-Fox 2012; Owens et al. 2020). The evolution of more detectable signals could, at least partly, mitigate the negative effect of artificial light on mate attraction. However, whether sexual selection for signal conspicuousness will result in an evolutionary response depends on the heritability of the signal and the factors that constrain signal evolution. These include physiological and morphological limitations, costs of signaling, and trade-offs in allocation of energy to different traits (Andersson 1994; Jennions et al. 2001).

We investigated whether artificial light alters sexual selection on signal intensity, in this case glow brightness, in the European common glow-worm *Lampyris noctiluca*. To attract flying males, flightless females emit a continuous cold light from a lantern on the underside of their abdomen. Females benefit from mating rapidly because they only have a limited amount of available resources after emerging as adults and lose eggs each day until they mate (Wing 1989). Females with a brighter glow are quicker to attract a male (Hopkins et al. 2015; Lehtonen and Kaitala 2020), and signal brightness correlates with body size (Hopkins et al. 2015). Here, we assessed, in a field experiment, the effects of three different intensities of artificial light, control (0.1–0.6 lux, *N* = 21), intermediate, (7–10 lux, *N* = 23), and high (16–20 lux, *N* = 20) (Figure 1), on glow-worm female mate attraction success (see Supplementary Material for additional information). The artificial light levels were chosen to mimic those of low- to medium-intensity street lights at the street level, with typical values ranging between 10 and 60 lux. The intensity of typical moonlight, in turn, is only 0.05–0.1 lux (Kyba et al. 2017). Two rivalling signalers, i.e., dummy females that were designed to trap males attracted to them, were placed at an equal distance from the source of light (Figure 1A). The two dummy females differed in signal brightness, with peak glow intensities of 0.016 µW/nm and 0.13 µW/nm, mimicking a dim and a very bright wild female, respectively (Hopkins et al. 2015; Lehtonen and Kaitala 2020; unpublished spectrophotometer data from 56 wild females by A-M Borshagovski). The experiment was performed at 4 sites, resulting in 4 replicates per night, with the treatments rotating among the sites (see Supplementary Material for further methodological details).

**Figure 1.**
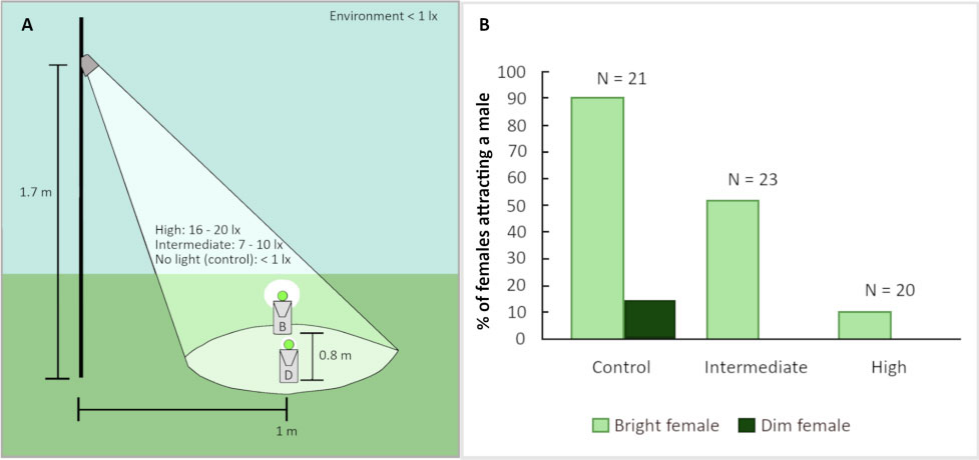
(A) The design of the field experiment with a bright (B) and a dim (D) dummy female in each replicate. The height of the pole with an artificial light source (LED light), the distances between the two dummies and between the dummies and the artificial light source, and the intensities of artificial light in the three treatments are indicated. (B) The percentage of B and D dummy glow-worm females successfully attracting at least one male. The number of replicates in each of the three artificial light treatments is also given.

The interaction term between dummy brightness and intensity of artificial light was nonsignificant and removed from the model (generalized linear mixed model: $\chi^{2}{}_{2}=\;0.2601$, *P* = 0.88). The refitted model showed that the probability of a dummy female attracting males depended on both its brightness (χ^2^_1_= 51.96, *P* < 0.001) and the intensity of the artificial light ($\chi^{2}{}_{2}=\;35.39$, *P* < 0.001): the brighter dummy female was more likely to attract males, and the likelihood of successful mate attraction decreased with artificial light intensity (Figure 1B and Supplementary Table S1). Both artificial light intensities reduced mate attraction success compared to the control (intermediate light intensity: *Z* = −2.731, *P* = 0.017, high light intensity: *Z* = −3.972, *P* < 0.001; Supplementary Table S1). The high light intensity had a stronger negative effect than the intermediate light intensity (*Z* = −2.441, *P* = 0.038; Figure 1B and Supplementary Table S1). The dimmer dummy female did not attract any males in the presence of artificial light (Figure 1B).

Our results show that while females are less likely to attract males under artificial light, sexual selection for brighter signals nevertheless continues to operate. Hence, the results indicate that sexual selection has the potential to promote the evolution of brighter signals under artificial light. The negative effect of artificial light on female mate attraction is in line with earlier findings on effects of street lights on mate attraction in Lampyrids (Bird and Parker 2014; Elgert et al. 2020). The results also show that the negative effect increases with the intensity of artificial light as demonstrated by the diminishing success of the brighter dummy females (Figure 1B). Interestingly, the dimmer females attracted no males in the presence of artificial light. This could be because males either actively selected the brighter of the two females or because they failed to detect the dimmer female and, hence, passively selected the brighter one.

Overall, our study shows that the negative impact of artificial light on glow-worm mate attraction increases with the intensity of artificial light, but sexual selection for brighter signals nevertheless prevails. If the selection results in an evolutionary response, it would mitigate the negative effect of light pollution on mating success. However, several factors could restrict an evolutionary response. The costs of signals, for instance those arising from signal production and predation risk, are likely to increase with brightness, which could constrain the evolution of brighter signals. In addition, signal brightness correlates positively with body size (Hopkins et al. 2015), and an increase in signal brightness may need to be traded-off against other fitness-related traits, such as shorter larval development time. The heritability of the signal, and thus the potential for evolutionary change, is not known. More research is therefore needed on the factors that constrain evolutionary responses to artificial light. In this respect, our results on the sexual selection for brighter signals prevailing under artificial light build a foundation for further studies on the mechanisms that promote or hinder adaptation to light pollution in the common glow-worm.

## Supplementary Material

zoaa071_Supplementary_Data
