# Supplementary material for: Sexual selection for bright females prevails under light pollution
Source: Curr Zool. 2020 Dec 28;67(3):329–31. doi: 10.1093/cz/zoaa071 (PMC8489004; doi:10.1093/cz/zoaa071)
Supplement: zoaa071_Supplementary_Data [file zoaa071_supplementary_data.zip › Elgert_Supplementary_Material.pdf]

Sexual selection for bright females prevails under light pollution

Christina Elgert[1,3], Topi K. Lehtonen[1, 2,3], Arja Kaitala[2,3] and Ulrika Candolin[1,3]

[1] Organismal and Evolutionary Biology, University of Helsinki, PO Box 65, 00014 Helsinki, Finland

[2] Department of Ecology and Genetics, University of Oulu, PO Box 3000, 90014 Oulu, Finland

[3]Tvärminne Zoological Station, University of Helsinki, J.A. Palménin tie 260, 10900 Hanko, Finland

Correspondence

Christina Elgert: christina.elgert@helsinki.fi

Supplementary Information

Methods

We performed the experiment in the field in the vicinity of Tvärminne Zoological Station in southern Finland (59°50.7´ N; 23°14.9´ E). The experiment was conducted during the peak breeding season of the glow-worm, from June to early July in 2019. We assessed the effects of three different intensities of artificial light, control, intermediate, and high, on mate attraction success of two competing dummy females (bright and dim) in each replicate. Light intensities of the dummy females and artificial light used in this study were measured with an Ocean Optics Flame – S miniature spectrometer combined with an integrating sphere for the dummy females, and a cosine corrector for environmental values. An AIRAM UVM-8 lx-meter was used to estimate the lx-values.

The dummy females were designed to trap males attracted to light akin to that emitted by live females. In particular, each dummy consisted of a green 5 mm LED-light at the top of a funnel trap (see Figure S1 and Lehtonen and Kaitala 2020 for construction). The wavelength of the LED-light was

562 nm, similar to the glow of female glow-worms (550 – 570 nm) (De Cock 2004; Bird and Parker 2014). The brighter of the two dummy females in each replicate had a light intensity similar to that of a very bright wild female (peak glow intensity 0.13 µW/nm), and the dimmer dummy female had a light intensity similar to that of a dimmer wild female (peak intensity 0.016 µW/nm). Thus, the difference in brightness between the two dummies reflected the range seen in the field, based on spectrophotometer measurements on 56 wild females in 2017 and 2018 (A-M Borshagovski, 2017 – 2018, unpublished spectrophotometer measurements) and personal observations (C Elgert, 2017 – 2020). The distance between the two dummy females and the artificial light source was 1 m, and the distance between the two dummies was 0.8 m (Figure 1A). The distance between the two dummy females should have allowed flying males to judge the brightness difference between them (Hopkins 2018).

The artificial light source was a pole with an attached white LED light (ANSI FL1 Standard: 35 lumen, beam distance 24 m) at the height of 1.7 m (Figure 1B). We had three treatments that differred in intensity of artificial light, measured at the ground level: no artificial light (control: the LED switched off, environmental light values depending on weather conditions: 0.1 – 0.6 lx, peak intensity ~0.0003–0.0016 µW/cm^2/nm at 460 nm), intermediate light intensity (7 – 10 lx, peak intensity ~0.025 µW/cm2/nm at 455 nm) and high light intensity (16 – 20 lx, peak intensity ~0.06 µW/cm^2/nm at 455 nm). Both female dummies were located within the brightest part of the cone of light from the artificial light source. The artificial light levels were chosen to mimic those of low to medium intensity streetlights at the street level, with typical streetlighting ranging between 10 and 60 lx, depending on the type. The intensity of typical moonlight, in turn, is only 0.05 – 0.1 lx (Kyba et al. 2017).

At 22:00 – 23:00 when dusk began to fall, we initiated each replicate by turning on the light of the two dummy females and the light from the pole (in the intermediate and high light intensity treatments). We checked the dummy females ~4 h later at 02:00 – 02:30 and counted the number of

males trapped by each dummy female. We colour-marked the captured males and released them the following day. Males that were recaptured during subsequent nights (N = 2) were not included in the analyses. We recharged the batteries energising the dummy females every other day, and those of the artificial lights every day.

The experiment was performed at 4 sites (resulting in 4 replicates per night), and the treatments were rotated among the sites. The distance between the sites ranged from 150 to 550 meters. We conducted 21 replicates of the control, 23 replicates of the intermediate artificial light intensity, and 20 replicates of the high light intensity.

Statistical analysis

We used R 3.6.3 (https://www.r-project.org/) to analyse the data. We excluded nights when no males were caught at any of the four sites. To determine the impacts of dummy female brightness and artificial light intensity on the likelihood that a dummy female attracted a male, we used a generalized linear mixed model (GLMM) with a binomial distribution and a logit link function (Bolker et al. 2009). The response variable was the presence or absence of male(s) in the dummy female trap (rather than the number of attracted males) to rule out a potential effect of the first male attracting or repelling additional males. Because all replicates of some combinations of female brightness and light intensity had the same binary value (in this case 0), i.e. “complete separation” (see Heinze and Schemper 2002; Ghosh et al. 2018), we used the “bglmer” function from the “blme“ package (version 1.0-4, see Dorie 2015). This function is similar to “glmer” of the “lme4” package but allowed us to impose a zero-mean normal prior probability distribution on the fixed effects (a 6 × 6 diagonal variance-covariance matrix, for variances of 9 or standard deviations of 3, see Bolker 2015; 2018) and, thus, address the issue of complete separation without the need to remove any variables (Bolker 2015; Dorie 2015; Ghosh et al. 2018; Lemoine 2019). Fixed factors were female brightness (brighter, dimmer) and the intensity of artificial light (control, intermediate, high) and their interaction. Replicate ID was added as a random effect to account for observing two females in each

replicate. We assessed the significance of the interaction term using a χ^2 - test: if it was non-significant (P > 0.1), we refitted the model without the interaction term. We compared the pairwise effects of the different intensities of artificial light using Tukey's HSD and the "glht" function from the "multcomp" package version 1.4-12 (Hothorn et al. 2008; Hothorn et al. 2020).

References

Bird S, Parker J, 2014. Low levels of light pollution may block the ability of male glow-worms *(Lampyris noctiluca* L.) to locate females. *J Insect Conserv* **18**:737–743. (doi: 10.1007/s10841-014-9664-2).

Bolker BM, 2015. Linear and generalized linear mixed models. In: Fox GA, Negrete-Yankelevich S, Sosa VJ, editors*. Ecological Statistics: Contemporary theory and application*. 1st ed. Oxford, New York: Oxford University Press. p. 309–333.

Bolker BM. 2018. GLMM worked examples from chapter 13 in Ecological Statistics: Contemporary Theory and Application. Available from https://bbolker.github.io/mixedmodels-misc/ecostats_chap.html#digression-complete-separation accessed 24th July.

Bolker BM, Brooks ME, Clark CJ, Geange SW, Poulsen JR, et al., 2009. Generalized linear mixed models: a practical guide for ecology and evolution. *Trends Ecol Evol* **24**:127–135. (doi: 10.1016/j.tree.2008.10.008).

De Cock R, 2004. Larval and adult emission spectra of bioluminescence in three European firefly species. *Photochem Photobiol* **79**:339–342. (doi: 10.1562/2003-11-11-RA.1).

Dorie V. 2015. blme: Bayesian linear mixed-effects models, version 1.0-4. Available from http://cran.r-project.org/web/packages/blme/index.html, accessed 25th March 2020.

Ghosh J, Li Y, Mitra R, 2018. On the Use of Cauchy Prior Distributions for Bayesian Logistic Regression. *Bayesian Anal* **13**:359–383. (doi: 10.1214/17-BA1051).

Heinze G, Schemper M, 2002. A solution to the problem of separation in logistic regression. *Stat Med* **21**:2409–2419. (doi: 10.1002/sim.1047).

Hopkins J, 2018. The costs and consequences of female sexual signals. PhD. Acta Universitatis Ouluensis: University of Oulu.

Hothorn T, Bretz F, Westfall P, 2008. Simultaneous inference in general parametric models. *Biometrical J* **50**:346–363. (doi: 10.1002/bimj.200810425).

Hothorn T, Bretz F, Westfall P, Heiberger RM, Schuetzenmeister A, et al. 2020. Multcomp: simultaneous inference in general parametric models, version 1.4-12. Available from https://cran.r-project.org/web/packages/multcomp/multcomp.pdf accessed 25th March 2020.

Kyba C, Mohar A, Posch T, 2017. How bright is moonlight. *Astron Geophys* **58**:31–32.

Lehtonen TK, Kaitala A, 2020. Leave me alone: solitary females attract more mates in a nocturnal insect. *Behav Ecol* **31**:1040–1045. (doi: 10.1093/beheco/araa049).

Lemoine NP, 2019. Moving beyond noninformative priors: why and how to choose weakly informative priors in Bayesian analyses. *Oikos* **128**:912–928. (doi: 10.1111/oik.05985).

Supplementary table

Table S1 Results of the GLMM. The response variable was the presence or absence (0/1) of males. Fixed factors were female brightness (brighter, dimmer) and the intensity of artificial light (control, intermediate, high) and their interaction. Replicate ID was denoted as a random effect. After the interaction term was found non-significant using a χ^2 - test ($P > 0.1$) (upper panel), the model was refitted without it (middle panel). The pairwise comparisons of the artificial light intensities were done using Tukey's HSD (lower panel)

**GLMM, interaction**	**χ2**	**df**	**P**
**Female brightness*Artificial light**	0.2601	2	0.88

**Refitted GLMM**	**χ2**	**df**	**P**
**Female brightness**	51.96	1	**< 0.001**
**Artificial light**	35.39	2	**< 0.001**

**Pairwise comparisons of artificial light levels, Tukey's HSD**	**Z**	**P**
**Intermediate light vs. Control**	-2.731	**0.017**
**High light vs. Control**	-3.972	**< 0.001**
**High light vs. Intermediate light**	-2.441	**0.038**
